# A randomized controlled trial on mobile phone text messaging to improve sexo-reproductive health among adolescent girls in Cameroon

**DOI:** 10.1186/s40834-022-00180-1

**Published:** 2022-07-03

**Authors:** Frankline Sevidzem Wirsiy, Catherine Atuhaire, Joseph Ngonzi, Samuel Nambile Cumber

**Affiliations:** 1 United Nations Office for the Coordination of Humanitarian Affairs, Yaoundé, Cameroon; 2 United Nations Volunteer, Bafoussam, Cameroon; 3 Department of Public Health and Hygiene, Faculty of Health Sciences, University of Buea, Buea, Cameroon; 4 Faculty of Medicine, Department of Nursing, Mbarara University of Science and Technology, Mbarara, Uganda; 5 Faculty of Medicine, Department of Obstetrics and Gynaecology, Mbarara University of Science and Technology, Mbarara, Uganda; 6 Centre for Health Systems Research & Development, University of the Free State, Bloemfontein, South Africa; 7 Office of the Dean, Faculty of Health Sciences, University of the Free State, Bloemfontein, South Africa; 8 School of Health Systems and Public Health, Faculty of Health Sciences, University of Pretoria, Pretoria, South Africa; 9 Institute of Health and Care Sciences, The Sahlgrenska Academy at University of Gothenburg, Gothenburg, Sweden

**Keywords:** Mobile phone, Sexo-reproductive health, Improved perception, Adolescent girls

## Abstract

**Background:**

We conducted a single-centered randomized controlled single-blinded trial (i.e. trained interviewers; blinded to group allocation). The target population included adolescent girls in the Kumbo West Health District (KWHD) of Cameroon. This trial tested the efficacy of weekly educational one-way text messages to improve perception of adolescent girls on sexo-reproductive health.

**Methods:**

Allocation concealment (1:1) was determined by sequentially numbered sealed opaque envelopes. A total of 398 participants either received the mobile phone sexo-reproductive health text messages (199) or not (199). A blinded program secretary send out text messages and recorded delivery. Data was collected and managed at baseline and at 6 month intervals using an interviewer-administered questionnaire before and after intervention, then analysed using the independent T-test (mean differences) and ANOVA on SPSS version 21.

**Results:**

The mean knowledge, attitude and practice scores respectively increased significantly from 6.03, 4.01 and 3.45 at baseline to 7.99, 5.83 and 4.99 at the end of the study. After performing ANOVA for the overall correct knowledge, positive attitudes and good practices respectively for between and within the intervention groups, we obtained: (F = 15.12, *P* = 0.023), (F = 60.21, *P* = 0.001) and (F = 57., *P* = 0.013) which showed statistical significance thus indicating the overall improvement in adolescents girls perception as a result of the intervention and not by chance. Majority (65.3%) of the participants were satisfied with the Short Message Service (SMS).

**Conclusion:**

This trial has contributed to the body of knowledge and evidence on the use of mobile phone technology using educative SMS to improve adolescent girl’s perception on sexo-reproductive health in Cameroon.

**Trial registration:**

Pan African Clinical Trials Registry, PACTR201805003259293. Registered 28 March 2018.

## Introduction

Adolescent girls in sub-Saharan Africa face various sexo-reproductive health risks such as unplanned pregnancy and sexually-transmitted infections (STIs), including human immunodeficiency virus (HIV) infections [1]. Adolescent girls are less likely than older women to access sexo-reproductive health care, including modern contraception and skilled assistance during pregnancy and childbirth [2]. Many are poor, have little control over household income, limited knowledge about sexo-reproductive health issues, and lack the ability to make independent decisions about their health [3]. Moreover, they often do not have access to health care that meets their specific sexo-reproductive health needs (SRHN). Most importantly, adolescent girls sexo-reproductive health needs often go unnoticed or are viewed through the lens of religious and cultural values, which in turn limit the possibility to provide highly needed care [4]. In Sub-Saharan Africa, few interventions have examined digital health solutions to adolescent sexo-reproductive health [5] (most often they are online based and hardly reach disadvantaged local communities) but yet none have primarily focused exclusively on using offline mobile phone short message service (SMS) interventions; that will create a larger impact [6]. Mobile text messages using the short message service (SMS) are a cheap and non-invasive means of communication that can be used to convey health related messages to mobile phone users [7]. The World Health Organization (WHO) has prioritized the use of new technologies to assist healthcare delivery in resource-limited settings [8]. For many adolescents especially girls in developing countries, the mere onset of puberty that occurs during adolescence marks a time of heightened vulnerability—to early pregnancy, HIV, leaving school, child marriage, sexual exploitation, coercion and violence [9]. Text-messaging programs have been shown to improve adolescents reproductive health knowledge and had the potential to lower pregnancy risk for sexually active adolescent girls [5, 10]. Another study reported high satisfaction with two way text messaging [11]. These findings suggest that the more feasible application of the mobile phone in health would be the SMS. Equally, on our search for papers on mobile phone programs for adolescent sexual and reproductive health in low- and middle-income countries, we found out that most projects (70%) relied on text messaging/short message service (SMS) to transmit sexo-reproductive health information to their users. These programs demonstrated the wide utility of SMS as a way to transmit and facilitate knowledge sharing within varied domains of adolescent SRH. Programs such as OneWorld and Education as a Vaccine (EVA) in Nigeria [12], mCENAS in Mozambique [13], M-ASSIST [14] and Project Khuluma [15] in South Africa, highlighted the various ways in which SMS has been leveraged to transmit information, support, and counseling on different SRH services. For example, In Nigeria, My Question—OneWorld and Education as a Vaccine (EVA) used SMS to transmit messages on SRH information and education delivery service for young adults. Text message interventions are capable of producing positive change in preventive health behaviors [16]. The goal of this trial was to assess whether sending weekly sexo-reproductive health text messages via a mobile phone versus no text messaging will improve adolescents girls perception on sexo-reproductive health; specifically to improve knowledge of reproductive health, ever-use of contraceptive methods, HIV/AIDS and sexually transmitted diseases, condom knowledge and attitudes, use and perceptions of health services among adolescent girls. The Mobile adolescent sexo-reproductive health scheme (MASHS) trial is registered with the Pan-African Clinical Trials Registry (http://pactr.org) as PACTR201805003259293.

## Methods

### Study design

This was a randomized control trial. MASHS was implemented by a before-and-after intervention strategy which involved the random selection of the intervention and control groups obtained through cluster sampling such that for the intervention group, we used a unique SMS update portal named MASHS. This consisted of the main operator and the beneficiary who was in possession of a mobile phone. The operator generated the content of clear and understandable messages based on key variables of adolescent sexo-reproductive health.

### Study procedures

**Fig. 1:**
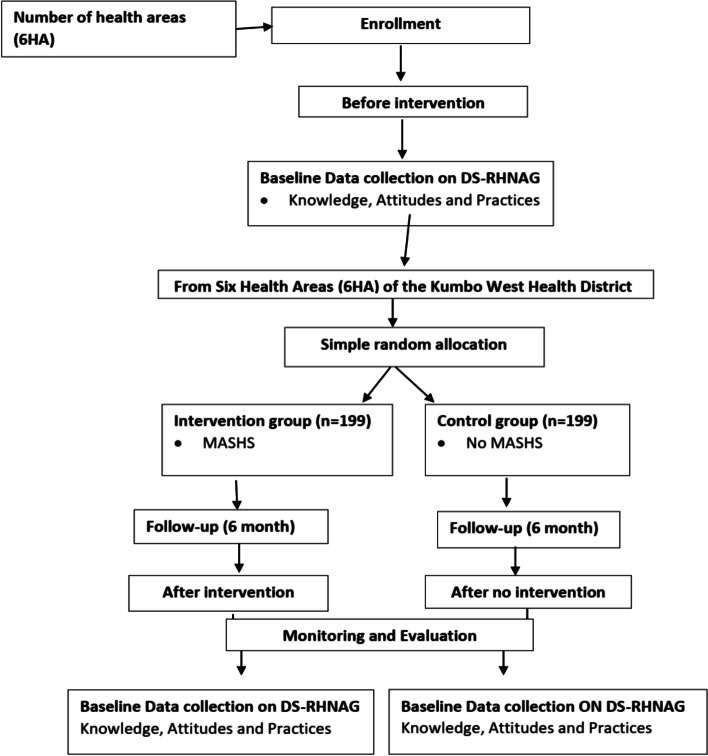
Flow chart of Mobile-based Adolescent Sexo-reproductive Health Scheme (MASHS) trial design, Kumbo West Health District 2018. HA- Health Area, DS-RHNAG-Determinants of ¨Sexo-Reproductive¨ Health Needs of Adolescent Girls

To start the intervention, MASHS was introduced and explained to the families of participants. They expected three to five messages from the operator per week for a period of 6 months. Ethical and administrative considerations were highly considered. Using a 1:1 allocation concealment ratio, adolescent girls in Kumbo West Health District were randomized to receive a one-way text message on sexo-reproductive health or not. Figure 1 shows a flow chart of MASHS study design which was in accordance with the guidelines of the Standard Protocol Items: Recommendations for interventional Trials (SPIRIT).

### Sample size determination

The sample size calculation for this trial study will be based on sample size calculation for comparison between two groups [17]as follows,

$$n=\frac{2({{z}_{\alpha /2}+ {z}_{\beta })}^{2} p(1-p)}{({{p}_{1}-{p}_{2})}^{2}}$$

$$n=\frac{2({1.96+ 0.84)}^{2} 0.43(1-0.43)}{({0.48-0.38)}^{2}}=384$$

${z}_{\alpha /2}$=z_0.05/2_ = 1.96 (from z table or type 1 error of 5%).

z_β/2_ = z_0.20_ = 0.842 (from z table at 80%).

p_1_-p_2_ = pooled prevalence = (prevalence in intervention group (p_1_) + prevalence in control group (p_2_)/2

$$n=384$$

Considering non-response, we rounded up to *n* = 398.

Hence, 199 subjects for the control group and 199 subjects for the intervention group.

### Sampling

**Fig. 2:**
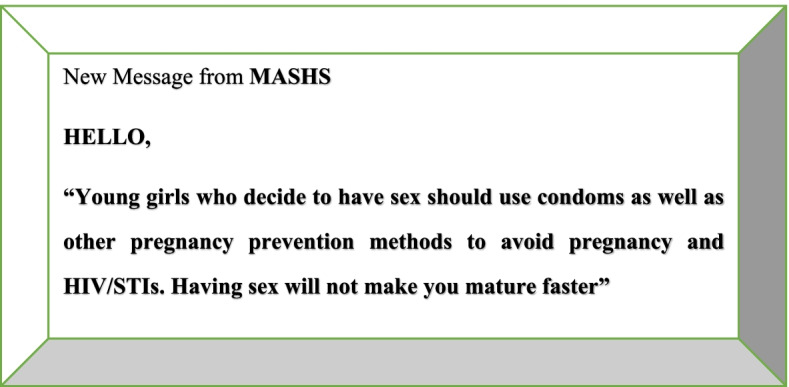
Example of a text message used in the intervention phase, MASHS 2018

Multi-stage cluster sampling was used to select 6 health areas from the Kumbo West Health District (See Fig. 2 showing the map of Kumbo West Health District-KWHD) namely BBH, Kikaikelaiki, Kitiwum, Kumbo_CMA, Kumbo_Urban and Melim in the Bui division of the North-West region of Cameroon and the participants were further selected using systematic random sampling from households. The various participants were then obtained via probability proportionate to size from the respective selected health areas.

### Simple random allocation of participants

This was a parallel group design evaluating the effects of adding weekly one-way SMS messages using mobile phones to usual care (intervention) versus usual care alone (control) among adolescent girls. Eligible and consenting girls were then randomized to intervention and control arms using a 1:1 allocation concelament ratio by opaque sealed envelope method. A computer generated randomization list was generated using random block sizes of 2, 4 and 6 to prevent selection bias and enabled the production of the comparable groups for this trial. The allocation codes were then put in sequentially numbered opaque sealed envelopes and administered by the trained research staff at the office of KWHD services.

### Training of interviewers

The interviewers were trained using the adult learning theory for three days. These trained interviewers; blinded to group allocation, collected baseline data using a pretested data collection form containing socio-demographic data, sources of information on, and knowledge of reproductive health, knowledge and ever-use of contraceptive methods, knowledge of HIV/AIDS and sexually transmitted diseases, condom knowledge and attitudes, use and perceptions of health services at baseline and at 6 month intervals. The data analyst was also blinded to group allocation.

### Trial setting

Cameroon is a culturally diverse coastal country in Africa, which lies on the western side of Africa on the Eastern Atlantic Ocean [18]. Cameroon is bordered by Chad, Nigeria, the Central African Republic, Gabon, Equatorial Guinea, and the Republic of the Congo. The 2018 Cameroon population is estimated at 24.68 million [18]. The study was conducted in Kumbo located in the North West region of Cameroon. The NorthWest Region is the third most populated region in Cameroon with Bamenda as capital [19]. This study was precisely carried out in the Kumbo West Health District (KWHD), a semi-urban/rural community with many adolescent girls. BBH, Kumbo-Urban and Kumbo-CMA are semi-urban areas while Melim, Kitiwum and Kikaikelaiki are rural areas. The study involved community mobilization and participatory approaches which specifically were implemented in six randomly selected health areas of the KWHD. According to the Department of Economic and Social Affairs; Population Division of the United Nations, 12.3 and 10.5 percent [20] of the total population make up the early adolescent girls (10–14 years) and late adolescent girls (15–19 years) population in Cameroon respectively. This gives a total of 22.8 percent adolescent girls of the total population of KWHD. Applying these population model we have a total of 25,120 adolescent girls in the KWHD. The trial ran for six months, with outcome assessment at baseline and at 6 month intervals.

### Participants: Inclusion/exclusion criteria

We included adolescent girls aged 10–19 years. We send the text messages to the adolescent girls or the parents/guardians of the adolescent girl that owned a mobile phone and could read text messages. Informed assent and consent was a prerequisite for participating in the study, and was provided orally and in writing. We excluded participants who refused to participate in the study and those aged less than 10 years or above 19 years.

### Intervention: Mobile-based adolescent sexo-reproductive health scheme (MASHS)

We sent a one-way short text message to the participants in the intervention group in English. These messages were sent three to five times per week for a period of 6 months. We sent the messages at afternoon periods on Mondays, Wednesdays and Fridays between 4-6 pm on those days. The content of the messages were motivating and acted both as a reminder and a cue to action (Fig. 2: show an example of text messages used in the intervention phase). The message was sent through a phone number they could call back or text if they needed clarifications. The content was varied so as to retain participants' attention throughout the period of the study. The program secretary had a list of phone numbers to which he send the messages every week and used the 'delivery report' function to ensure that the messages had been delivered. The average cost for text messages on any networks in Cameroon is 50 CFA Frs.

### Control

In the control arm, subjects received the usual care provided to all adolescent girls as concerns sexo-reproductive health education which included education at schools and reproductive health services delivered at the health facilities of KWHD. They did not receive any text messages, but were interviewed at baseline and 6 months.

### Administration of questionnaires at before and after intervention

A structured questionnaire was administered to selected adolescent girls who agreed to participate in the study. The questionnaires were interviewed-administered in English/Pidgin- English. The questionnaire was made up of four sections namely, i. Socio-Demographic Information, ii. Knowledge related questions on sexo-reproductive health iii. Attitude related questions on sexo-reproductive health and iv, Practice related questions on sexo-reproductive health.

### Data management and analysis

A coded number was given to identify each participant. Collected data was firstly entered into a research log book. Research questionnaires as well as work books and other study materials were stored safely in a locker in a safe location and secured by locking it with a lock. After collection of the data, the questionnaires were checked visually for completeness, obvious errors, and inconsistencies and then corrected. Data collected was entered daily into an electronic questionnaire (template) created in Epi info version 7 by the investigator. During the data entry process, 10% of data entered at the beginning was checked to ensure that the data was correctly entered. For confidentiality, the computer in which the data was stored was password protected and the information was accessible only to the researcher. Data was backed-up in an external hard drive and email box. The data was then imported into Microsoft excel spread sheet for cleaning/editing and finally analysed using social science package statistical software version 21. Descriptive and analytic statistics were performed.

### Ethical considerations

The trial was conducted in compliance with the local protocol and applicable regulatory requirements in Cameroon. The study was approved by the Institutional Review Board of the Faculty of Health Sciences of the University of Buea (2018/193/UB/SG/IRB/FHS). Any deviations from the protocol were reported and explained. The study was conducted in accordance with the Helsinki declaration [21] and other established clinical practice guidelines for research on human subjects. Research personnel approached all potentially eligible adolescent girls who fulfilled eligibility criteria for assent/consent. All adolescents’ girls signed a consent form or gave verbal consent to participate in the trial. Its Pan-African Clinical Trials Registry number is PACTR201805003259293.

## Results

### Socio-demographics characteristics of participants in the intervention and control groups of the trial

Data from 398 randomized participants (adolescent girls) was analysed. This is such that we had 199 participants for control and 199 participants for the intervention. There was a homogeneous distribution of participants in both groups. Table 1 shows the socio-demographic characteristics of the participants in control and intervention groups for the RCT in KWHD, 2018. The two groups were similar in terms of socio-demographic characteristics such as age, education, religion and number of years expected to school at baseline.

**Table 1:** Sociodemographic characteristics of the participants in the intervention and control groups, Kumbo West Health District 2018

Characteristic	SMS Intervention (*n* = 199), No (%)	No SMS Intervention (*n* = 199), No (%)	χ ^2^	*P* value
**Age (years)**				
10 – 14	84 (42.2)	79 (39.7)		
15 – 19	115 (57.8)	120 (60.3)	0.251	**0.165**
**Total**	**199 (100)**	**199 (100)**		
**Education**				
No formal education	25 (12.6)	27 (13.6)		
Primary	114 (57.3)	118 (59.3)		
Secondary	60 (30.2)	54 (27.1)	0.036	**0.896**
**Total**	**199 (100)**	**199 (100)**		
**Religion**				
None	7 (3.5)	11 (5.5)		
Christian	169 (85.0)	159 (79.9)		
Muslim	23 (11.6)	29 (14.6)	0.243	**0.223**
**Total**	**199 (100)**	**199 (100)**		
**Living with parents**				
Yes	172 (86.4)	175 (87.9)		
No	27 (13.6)	24 (12.1)	0.039	**0.984**
**Total**	**199 (100)**	**199 (100)**		
**Number of years expected to continue schooling**				
1 – 7	74 (37.2)	70 (35.2)		
8 – 14	125 (62.8)	129 (64.8)	0.211	**0.751**
**Total**	**199 (100)**	**199 (100)**		
**Consumed alcohol in the last 30 days**				
Yes	28 (14.0)	37 (18.6)		
No	171 (86.0)	162 (81.4)	0.176	**0.662**
**Total**	**199 (100)**	**199 (100)**		

### Baseline data on knowledge, attitude and practice (KAP) of adolescent girls on sexo-reproductive health

Table 2 shows the baseline data regarding KAP of adolescent girls on sexo-reproductive health in the control and intervention groups. No significant difference was found in the knowledge, attitudes and practices of adolescent girls on sexo-reproductive health.

**Table 2:** Baseline data on knowledge, attitude and practice of adolescent girls on Sexo-reproductive Health, Kumbo West Health District 2018

Characteristic	SMS Intervention No (%)	No SMS Intervention No (%)	Mean diff
**Correct knowledge**			
Correct source of information on SRH	82 (41.2)	84 (42.2)	-1.000
Importance/benefits of abstinence to sex	165 (82.9)	166 (83.4)	-0.500
Time of sexual debut	111 (55.8)	114 (57.3)	-1.500
Number of sexual partners	173 (86.9)	170 (85.4)	1.500
Consequences of early pregnancy	183 (92.0)	183 (92.0)	0.000
Types and use of contraceptives	78 (39.2)	75 (37.7)	1.500
Prevention of HIV and other STIs	115 (57.8)	113 (56.8)	1.000
Consensual sex and sexual rights	111 (55.8)	110 (55.3)	0.050
Consequences of abortion	147 (73.9)	143 (71.9)	2.000
**Positive attitudes**			
Discussing sexual education with parents/teachers	70 (35.2)	71 (35.7)	-0.500
Use of contraception	65 (32.7)	66 (33.2)	-0.500
Sexual rights and denouncing rape	100 (50.3)	111 (55.8)	-5.500
Use of sexual and reproductive health services	25 (12.6)	20 (10.0)	2.600
Multiple sexual partners	73 (36.7)	71 (35.7)	1.000
HIV testing attitudes	99 (49.7)	95 (47.7)	2.000
Abortion	147 (73.9)	140 (70.4)	3.500
Transactional sex	150 (75.4)	153 (76.9)	-1.500
**Good practices**			
Abstained from sexual intercourse	121 (60.8)	120 (60.3)	0.500
Had consensual sex	38 (19.1)	38 (19.1)	0.000
Had less than two sexual partners	30 (15.1)	28 (14.1)	1.000
Used a condom or any other contraceptive	31 (15.6)	30 (15.2)	0.400
Use of sexual and reproductive health services	75 (37.7)	77 (38.7)	-1.000
Pregnancy	13 (6.5)	11 (5.5)	1.000
HIV testing practices	29 (14.6)	28 (14.3)	0.300

Generally at baseline, the mean KAP score of adolescents on SRH did not show any significant difference in the control and intervention groups (Table 3).

**Table 3:** Baseline data on knowledge, attitude and practice score of adolescent girls on Sexo-reproductive health, Kumbo West Health District 2018

Characteristic	Intervention group mean score (SD)	Control group mean score (SD)	*P* value
**Knowledge score (/9)**	6.03 (1.22)	6.01 (1.04)	0.178
**Attitude score (/8)**	4.01 (0.73)	4.15 (0.76)	0.854
**Practice score (/7)**	3.45 (0.42)	3.40 (0.33)	0.924

*SD* Standard Deviation

### Change in knowledge, attitude and practice after MASHS intervention

Table 4 shows the change in knowledge, attitude and practice after MASHS intervention. The proportion of adolescent girls with good knowledge, attitude and practice increased significantly from baseline to post-intervention in the intervention group but not in the control group. Knowledge increased significantly from baseline to post-intervention in the following parameters: correct source of sexual education (41.2% to 50.3%), time of sexual debut (55.8% to 63.2%), types and use of contraceptives (39.2% to 44.6%), prevention of HIV and other STIs (57.8% to 63.1%), sexual right and consequences of abortion (73.9% to 81.0%). Attitude increased significantly from baseline to post-intervention in the following parameters: discussing sexual education with parents (35.2% to 41.0%), use of contraceptives (32.7% to 37.7%), multiple sexual partners (36.7% to 40.6%), HIV testing and transactional sex (75.4% to 83.2%). Practice increased significantly from baseline to post-intervention in the following parameters: abstinence (60.8% to 72.5%), use of any contraceptive (15.6% to 19.1%) and HIV testing (14.8% to 20.3%).

**Table 4:** Change in knowledge, attitude and practice of adolescent girls on Sexo-reproductive health, Kumbo West Health District 2018

Characteristic	Intervention group (*n* = 199)			Control group (*n* = 199)		
	**Baseline %**	**Post-intervention %**	**Difference (*p*-value)**	**Baseline %**	**Post-intervention %**	**Difference (*p*-value)**
**Correct knowledge**						
Source of information on SRHN	41.2	50.3	9.1(0.014) ^*^	42.2	41.2	-1.0(0.665)
Benefits of abstinence to sex	82.9	85.6	2.7(0.214)	83.4	82.9	-0.5(0.984)
Time of sexual debut	55.8	63.2	7.4(0.014) ^*^	57.3	57.8	0.5 (0.847)
Number of sexual partners	86.9	87.3	0.4(0.698)	85.4	86.9	1.5(0.321)
Consequences of early pregnancy	92.0	93.0	1.0(0.69)	92.0	92.5	0.5(0.354)
Types and use of contraceptives	39.2	44.6	5.4(0.031) ^*^	37.7	39.2	1.5(0.112)
Prevention of HIV and other STIs	57.8	63.1	5.3(0.022) ^*^	56.8	57.8	1.0(0.565)
Concensual sex and sexual rights	55.8	63.0	7.2(0.013) ^*^	55.3	56.8	1.5(0.554)
Consequences of abortion	73.9	81.0	7.1(0.011) ^*^	71.9	73.9	2.0(0.112)
**Positive attitudes**						
Discussing sexual education with parents	35.2	41.0	5.8(0.013) ^*^	35.7	35.2	-0.5(0.851)
Use of contraception	32.7	37.7	5.0(0.0124) ^*^	33.2	32.7	-0.5(0.654)
Sexual rights and denouncing rape	50.3	50.3	0.0(1.000)	55.8	56.3	0.5(0.654)
Use of reproductive health services	12.6	13.5	0.9(0.478)	10.0	12.6	2.6(0.214)
Multiple sexual partners	36.7	40.6	3.9(0.033) ^*^	35.7	35.7	0.0(1.000)
HIV testing attitudes	49.7	59.2	9.5(0.011) ^*^	47.7	49.7	2.0(0.123)
Abortion	73.9	74.0	0.1(0.689)	70.4	72.9	2.5(0.211)
Transactional sex	75.4	83.2	7.8(0.014) ^*^	76.9	75.4	-0.5(0.144)
**Good practices**						
Abstained from sexual intercourse	60.8	72.5	11.7(< 0.001 ^*^	60.3	60.8	0.5(0.231)
Had consensual sex	19.1	20.4	1.3(0.365)	19.1	19.1	0.0(1.000)
Had less than two sexual partners	15.1	16.3	1.2(0.217)	14.1	15.1	1.0(0.235)
Used of any contraceptive	15.6	19.1	3.5(0.022) ^*^	15.1	15.6	0.5(0.365)
Use of SRH services	37.7	38.7	1.0(0.698)	38.7	35.2	-3.5(0.089)
Pregnancy	6.5	5.4	-1.1(0.54)	5.5	5.5	0.0(1.000)
HIV testing practices	14.6	20.3	5.7(0.012) ^*^	14.1	14.6	0.5(0.654)

Generally, after the intervention; the mean knowledge, attitude and practice scores increased significantly in the intervention group but not in the control group. The mean knowledge score increased significantly from 6.03 at baseline to 7.99 at the end of the study (Mean difference = 1.96, *P* < 0.001) in the intervention group. In the intervention group, attitude score was found to increase significantly from an average of 4.01 at baseline to 5.83 at the end of the study (Mean difference = 1.82, *P* = 0.021). In the intervention group, practice score was found to increase significantly from an average of 3.45 at baseline to 4.99 at the end of the study (Mean difference = 1.54, *P* = 0.041) as depicted on Table 5.

**Table 5:** Mean difference in knowledge, attitude and practice of adolescent girls on Sexo-reproductive health, Kumbo West Health District 2018

Characteristic	Intervention group (*n* = 199)			Control group (*n* = 199)		
	**Baseline Mean (SD)**	**Post-intervention Mean (SD)**	**Mean difference (*p*-value)**	**Baseline Mean (SD)**	**Post-intervention Mean (SD)**	**Mean difference (*p*-value)**
**Knowledge (/9)**	6.03 (1.22)	7.99 (1.23)	1.96(0.001) ^*^	6.01 (1.04)	6.02 (1.05)	0.01(0.221)
**Attitude (/8)**	4.01 (0.73)	5.83 (0.99)	1.82(0.021) ^*^	4.15 (0.76)	4.04 (0.65)	-0.11(0.547)
**Practice (/7)**	3.45 (0.42)	4.99 (0.43)	1.54(0.041) ^*^	3.40 (0.33)	3.41 (0.41)	0.01(0.232)

### Summary statistics; Analysis of variance on correct knowledge, positive attitudes and good practice of adolescent girls on sexo-reproductive health in the Kumbo West Health District

To determine whether any of the differences between the means of the knowledge, attitudes and practices were statistically significant, we performed an Analysis of variance test and as such compared the p-value to our significance level (*p* ≤ 0.05). Equally, in the ANOVA test, the p values were determined by the F statistic (the probability that our results could have happened by chance). A large F ratio meant that the variation among group means is more than you'd expect to see by chance. It is in this light that, inorder to assess the impact of our intervention, we performed ANOVA test that indicated the intervention doped Mobile Adolescent sexo-reproductive health scheme (MASHS) had an influence on adolescent girls improvement on overall perception on sexo-reproductive health at post intervention. Table 6 and 7 shows summary statistics of correct knowledge before and after intervention on adolescent girl’s sexo-reproductive health in the 6 health areas randomly selected for the RCT in the Kumbo West Health District (KWHD) of Cameroon. In Table 6, before the intervention, performing the ANOVA test on overall correct knowledge for between and within the intervention groups, the *P* = 0.179 showed no statistical significance.

**Table 6:** Summary statistics of correct knowledge on Sexo-reproductive health before intervention among adolescent girls in the 6 different health areas of the Kumbo West Health District, 2018

Summary statistics of correct knowledge on SRHN before intervention					
**Health area**	**Sample size**	**Total score**	**Mean score**	**Variance**	**Standard deviation**
BBH	117	51	5.70	4.31	1.31
Kikaikelaiki	39	38	4.32	3.04	1.16
Kitiwum	30	34	4.11	2.44	1.01
Kumbo CMA	106	46	5.15	1.15	1.33
Kumbo Urban	77	52	6.04	2.16	1.01
Melim	29	41	4.82	2.44	1.61
**Analysis of variance for correct knowledge on SRHN before intervention**					
Variation	Sum of square	Degree of freedom	Mean square	F-statistic	*p*-value
Between groups	109.8	5	21.96		
Within groups	1352.49	392	3.45	6.37	0.179
Total	1462.29	397

**Table 7:** Summary statistics of correct knowledge on Sexo-reproductive health after intervention among adolescent girls in the 6 different health areas of the Kumbo West Health District, 2018

Summary statistics of correct knowledge of adolescent girls on SRHN after intervention					
**Health area**	**Sample size**	**Total score**	**Mean score**	**Variance**	**Standard deviation**
BBH	117	60	6.71	5.32	1.61
Kikaikelaiki	39	48	5.33	4.03	1.32
Kitiwum	30	46	5.10	3.45	1.02
Kumbo CMA	106	55	6.16	2.14	1.65
Kumbo Urban	77	63	7.05	3.14	2.01
Melim	29	52	5.81	3.45	1.61
**Analysis of variance for correct knowledge of adolescent girls on SRHN after intervention**					
Variation	Sum of square	Degree of freedom	Mean square	F-statistic	*p*-value
Between groups	267.6	5	53.52		
Within groups	1387.68	392	3.54	15.12	0.023
Total	1655.28	397			

Whereas, in Table 7, after the intervention, the mean score of correct knowledge was highest in the Kumbo_Urban (7.05) health area seconded by BBH (6.71). After performing the ANOVA test on overall correct knowledge for between and within the intervention groups ( i.e. Adolescents girls who received SMS and Adolescents girls who didn’t receive SMS), the *P* = 0.023 showed statistical significance thus indicating the improvement in correct knowledge was as a result of the intervention doped MASHS. This was supported by the fact that, the F value for within groups was 15.12 which is higher than 1.0, thus the difference in means on correct knowledge scores and its statistical significance obtained was as a result of the effect of the intervention.

In the same light, Table 8 and 9 shows summary statistics of positive attitudes before and after intervention of adolescent girls on sexo-reproductive health in the 6 health areas. In table 36, before the intervention, performing the ANOVA test on overall positive attitudes for between and within the intervention groups, the *P* = 0.858 showed no statistical significance.

**Table 8:** Summary statistics of positive attitudes on Sexo-reproductive health before intervention among adolescent girls in the 6 different health areas of the Kumbo West Health District, 2018

Summary statistics of positive attitudes on SRHN before intervention					
**Health area**	**Sample size**	**Total score**	**Mean score**	**Variance**	**Standard deviation**
BBH	117	25	3.32	2.12	0.11
Kikaikelaiki	39	23	3.01	2.03	0.03
Kitiwum	30	16	2.09	1.56	0.32
Kumbo CMA	106	24	3.15	2.02	0.22
Kumbo Urban	77	22	4.26	2.95	0.45
Melim	29	14	2.71	1.60	0.30
**Analysis of variance for positive attitudes on SRHN before intervention**					
Variation	Sum of square	Degree of freedom	Mean square	F-statistic	*p*-value
Between groups	359.6	5	71.92		
Within groups	568.86	392	1.45	49.60	0.858
Total	928.46	397

**Table 9:** Summary statistics of positive attitudes on Sexo-reproductive health after intervention among adolescent girls in the 6 different health areas of the Kumbo West Health District, 2018

Summary statistics of positive attitudes of adolescent girls sexo-reproductive health					
**Health area**	**Sample size**	**Total score**	**Mean score**	**Variance**	**Standard deviation**
BBH	117	34	4.31	3.11	1.01
Kikaikelaiki	39	32	4.02	3.01	1.03
Kitiwum	30	25	3.10	2.65	1.32
Kumbo CMA	106	33	4.16	3.01	0.45
Kumbo Urban	77	41	5.25	3.96	1.41
Melim	29	30	3.81	2.60	1.31
**Analysis of variance for positive attitudes of adolescent girls sexo-reproductive health**					
Variation	Sum of square	Degree of freedom	Mean square	F-statistic	*p*-value
Between groups	463.6	5	92.72		
Within groups	603.68	392	1.54	60.21	0.001
Total	1067.28	397			

After the intervention, performing the ANOVA test on overall positive attitudes (Table 9) for between and within the intervention groups, the *P* = 0.001 showed statistical significance thus indicating the intervention doped MASHS impacted a rise in positive attitudes of adolescents girls on sexo-reproductive health. This was supported by the fact that the F value for within groups was 60.21 which is large, thus indicating the difference in means of positive attitude scores and its statistical significance gotten was not by chance, but as a result of the effect of the intervention.

Equally, Table 10 and 11 shows summary statistics of good practice before and after intervention of adolescent girls on sexo-reproductive health in the 6 health areas of KWHD. In table 38, before the intervention, performing the ANOVA test on overall good practice for between and within the intervention groups, the *P* = 0.915 showed no statistical significance.

**Table 10:** Summary statistics of good practices on Sexo-reproductive health before the intervention among adolescent girls in the different health areas of the Kumbo West Health District, 2018

Summary statistics of good practices on SRHN before intervention					
**Health area**	**Sample size**	**Total score**	**Mean score**	**Variance**	**Standard deviation**
BBH	117	22	3.21	2.31	0.21
Kikaikelaiki	39	21	3.02	2.61	0.93
Kitiwum	30	22	3.10	1.65	0.12
Kumbo CMA	106	22	3.16	1.31	0.35
Kumbo Urban	77	37	5.25	4.06	0.31
Melim	29	20	2.81	1.20	0.30
**Analysis of variance for good practices on SRHN before intervention**					
Variation	Sum of square	Degree of freedom	Mean square	F-statistic	*p*-value
Between groups	411.7	5	82.34		
Within groups	599.6	392	1.53	53.82	0.915
Total	898.3	397

**Table 11:** Summary statistics of good practices on Sexo-reproductive health after intervention among adolescent girls in the different health areas of the Kumbo West Health District, 2018

Summary statistics of good practices on SRHN after intervention					
**Health area**	**Sample size**	**Total score**	**Mean score**	**Variance**	**Standard deviation**
BBH	117	22	3.21	2.31	0.21
Kikaikelaiki	39	21	3.02	2.61	0.93
Kitiwum	30	22	3.10	1.65	0.12
Kumbo CMA	106	22	3.16	1.31	0.35
Kumbo Urban	77	37	5.25	4.06	0.31
Melim	29	20	2.81	1.20	0.30
**Analysis of variance for good practices on SRHN after intervention**					
Variation	Sum of square	Degree of freedom	Mean square	F-statistic	*p*-value
Between groups	433.7	5	86.74		
Within groups	596.6	392	1.52	57.07	0.013
Total	898.3	397			

Moreover, From Table 11, after performing the ANOVA test on overall good practice for between and within the intervention groups, the *P* = 0.013 showed statistical significance thus indicating the improvement in good practices of adolescent girls on sexo-reproductive health was as a result of the intervention doped MASHS. Furthermore, the F value for within groups was 57.07 which is higher than 1.0, thus the difference in means on good practice scores and its statistical significance established was not by chance, but as a result of the effect of the intervention.

### Satisfaction with text massages among participants who received text massages

Table 12 describes the satisfaction of participants with respect to the text message-based intervention. As concerns the rating of the frequency of the text messages, majority (65.3%) of the participants were satisfied with the text messages. Regarding the content of the text messages, majority (69.8%) of the respondents were satisfied. In terms of respect of ethics, only 45.3% of the respondents were satisfied. Friday (75.4%) was the preferred day for receiving the text messages. Majority (85.4%) of the respondents agreed they will continue receiving these text messages.

**Table 12:** Satisfaction with text massages among adolescent girls who received text massages in Kumbo West Health District, 2018 (*n* = 199)

Parameter	No (%)
**How do you rate the frequency of the text messages**	
Satisfactory	130 (65.3)
Neutral	44 (22.1)
Unsatisfactory	25 (12.6)
Sub-total	199 (100)
**How would you rate the content text messages?**	
Satisfactory	139 (69.8)
Neutral	40 (20.1)
Unsatisfactory	20 (10.1)
Sub-total	199 (100)
**How do you rate the text message in the terms of respect of ethics (religion, tradition and others)**	
Satisfactory	90 (45.3)
Neutral	50 (25.1)
Unsatisfactory	59 (29.6)
Sub-total	199 (100)
**Preferred day of the sending the text message**	
Monday	20 (10.0)
Wednesday	25 (12.6)
Friday	150 (75.4)
Sub-total	199 (100)
**Do you want to continue receiving these text massages?**	
Yes	170 (85.4)
No	29 (14.6)
Sub-total	199 (100)
**Would you recommend to a friend?**	
Yes	160 (80.4)
No	39 (19.6)
Sub-total	199 (100)

## Discussion

MASHS is an unconventional solution which helped overcome unmet adolescent sexo-reproductive health barriers by providing adolescent girls with a sustainable, accurate, timely, and engaging information that improved on their perception towards sexo-reproductive health. MASHS also offered privacy in comparison with face-to-face meetings with health care providers, and provided young people with tailored and anonymous health information without stigma or judgement. The study has shown that the potential of mobile phone technology using SMS to improve the knowledge, attitudes and practices of adolescent girls on sexo-reproductive health. Such improved perception (knowledge, attitudes and practices) results in improved health outcomes among adolescent girls. This is a domain worth exploring, especially in this era of increased uptake and dependence on mobile phones. Studies investigating the use of text messages to improve adolescent sexual and reproductive health yielded varied results [5, 22–24] though the results of this study correlates with that of Ippoliti [6].

The implementation of this intervention contributed in the improvement of adolescent sexo-reproductive health as shown by analysis of data thus enhancing prevention of adolescent pregnancies in Cameroon. Mobile phones are inexpensive, portable, and accessible. The penetration of mobile phone networks in many low- and middle-income countries surpasses other infrastructure such as paved roads and electricity, and dwarfs fixed Internet deployment. The growing sophistication of these networks – offering higher and higher speeds of data transmission alongside cheaper and more powerful handsets are transforming the way health services and information are accessed, delivered, and managed [25]. This promising approach called mHealth, which uses mobile phones to improve adolescent health knowledge, behaviors and outcomes, will have advantages when used in health programming for young people.

Adolescents commonly report low SRH knowledge and risky sexual behaviors [5, 6] but they also face barriers to care such as provider bias and fear of stigma, refusal, and embarrassment in seeking information and services. Mobile phone solutions help overcome many of these barriers by providing accurate, timely, engaging information and appropriate care for highly sensitive adolescent sexo-reproductive health topics [5]. This has been the case of MASHS intervention. Mobile phones offer privacy in comparison with face-to-face meetings with health care providers, and they provide young people with tailored and anonymous health information without stigma or judgment [26]. Furthermore, young people are responsive to and excited about using new technologies for SRH promotion.

MHealth as a technical area has seen increasing interest and promise from both developed and developing countries. While published research on adolescent sexo-reproductive health from higher income countries on mHealth solutions for SRH is growing, there is much less documentation of SRH mHealth interventions for youth living in resource-poor settings [5]. This intervention centered on the Health Belief Model which is a component of the Health Promotion theory [27]. After the intervention, majority of participants (96.5%) reported they will want to continue receiving the text messages. This is important as they want to promote their health status to a better one. Galovotti et al. [28] succinctly describe the key components of an intervention aimed at bringing about a change in behavior.

The results of this study show that SMS adds value to an improvement of sexo-reproductive health among adolescent girls. It has been found in several studies that the use of SMS generally improves health outcomes in resource limited settings like Cameroon. This was exemplified by Nsagha et al. [29] in 2016 that SMS improved adherence to Anti-retroviral (ARV) medication in Cameroon. The study further established that key constraints which affect adhere to ARV medication can be addressed using SMS. Also, Mbuagbaw et al. [30] in 2015 had reported that text messaging interventions improve health outcomes in people living with HIV and other chronic diseases.

## Conclusion

This study has contributed to the growing body of knowledge and evidence on the use of mobile phone technology as a complementary strategy for strengthening health systems and achieving health-related goals oriented towards adolescent girls sexo-reproductive health based on the results of the trial in the Kumbo West Health District of Cameroon.

## Data Availability

Data Base will be kept for five years.
